# Supplementary material for: PEG/PPG-PDMS-Adamantane-Based Crosslinked Terpolymer Using the ROMP Technique to Prepare a Highly Permeable and CO2-Selective Polymer Membrane
Source: Polymers (Basel). 2020 Jul 27;12(8):1674. doi: 10.3390/polym12081674 (PMC7464022; doi:10.3390/polym12081674)
Supplement: Supplementary file 1 [file polymers-12-01674-s001.pdf]

Supplementary materials

PEG/PPG-PDMS-Adamantane-based Crosslinked Terpolymer Using the ROMP Technique to Prepare a Highly Permeable and CO_2-selective Polymer Membrane

Dongyoung Kim,[1,2,a] Iqubal Hossain,[1,2,a] Yeonho Kim,[2] Ook Choi[2] and Tae-Hyun Kim[1,2,*]

[1] Organic Material Synthesis Laboratory, Department of Chemistry, Incheon National University, 119 Academy-ro, Yeonsu-gu, Incheon, 22012, Republic of Korea
[2] Research Institute of Basic Sciences, Incheon National University, 119 Academy-ro, Yeonsu-gu, Incheon, 22012, Republic of Korea
[a] These authors contributed equally to this work
* Correspondence: tkim@inu.ac.kr (T.-H. Kim); Tel. +82-32-8358232.

S-1. Synthesis of norbornene-functionalized precursor molecules 3, 4, 5

*S-1-1. Synthesis of NB-PPG/PEG-NB (**3**)*

For the synthesis of NB-PPG/PEG-NB (**3**), 1.5 g of NB (**2**)(9.13 mmol) and 8.25 g of Jeffamine® ED-2003 (4.33 mmol), 30 mL of acetic acid were used. After following the general procedure, the brownish resultant solution was cooled to room temperature and acetic acid was evaporated. After that, the viscous solution was washed with diethyl ether, followed by keeping in refrigerator and repeated several times to remove unreacted reaction residue. The brownish solid was obtained after dried for 48 h under vacuum at room temperature. (brownish solid, yield 66.5 %); δ_H(400 MHz, $CDCl_3$) 6.26(4H, s, H_a), 4.42-4.32(2H, br signal, H_g), 4.03-3.26 (190H, br signal, $H_{f,j,k,l}$), 3.25-3.18 (4H, br signal, H_b), 2.62-2.54(4H, br signal, H_e), 1.50-1.38(4H, br signal, H_c), 1.31-1.22(6H, br signal, H_d), 1.14-0.98(15H, br signal, H_i); ATR-IR (cm^{-1}) 2880(C-H stretching), 1768(imide C=O asymmetric stretching), 1696(imide C=O symmetric stretching), 1464(CH_2 deformation), 1340(CH_2 deforamtion), 1100(C-O-C stretching)

*S-1-2. Synthesis of NB-PDMS-NB (**4**)*

For the synthesis of NB-PDMS-NB (**4**), 0.5 g of NB (**2**)(3.05 mmol) and 3.045 g of PDMS (1.22 mmol), 20 mL of acetic acid were used. After following the general procedure, the resultant solution was cooled to room temperature and acetic acid was evaporated. Then, colorless liquid was extracted several times with dichloromethane and distilled water, followed by drying over anhydrous $MgSO_4$. The organic layer was evaporated under reduced pressure. After that, some volume of n-hexane was pour into this organic layer and keep in the refrigerator until white precipitate didn't appear. n-Hexane was dried under reduced pressure and organic product was filtered by using PTFE syringe filter, followed by drying for 48 h under vacuum at room temperature. (transparent oil, yield 78.1 %); δ_H(400MHz, $CDCl_3$) 6.26(4H, s, H_a), 3.45-3.39(4H, t, *J*=8Hz, H_f), 3.26(4H, s, H_b), 2.65(4H, s, H_e), 1.60-1.46(6H, m, $H_{g,c}$), 1.22(2H, s, H_d), 0.50(4H, t, *J*=8Hz, H_h), 0.20~-0.10(182H, br, H_i); ATR-IR (cm^{-1}) 2962(C-H stretching), 2904(C-H stretching), 1772(imide C=O asymmetric stretching), 1704(imide C=O symmetric stretching), 1258(Si-CH_3 bending), 1078(Si-O-C stretching), 1010(Si-O-Si stretching), 788(Si-CH_3 rocking).

*S-1-3. Synthesis of NB-Ad (**5**)*

For the synthesis of NB-Ad (**5**), 1 g of NB (**2**)(6.09 mmol) and 1.842 g of 1-Adamantylamine (12.18 mmol), 7 mL of acetic acid were used. After following the general procedure, the resultant solution was cooled to room temperature and and add some volume of dichloromethane to dissolve solidified product, followed by evaporating acetic acid and dichloromethane. After that, the product was extracted several times with dichloromethane and distilled water. The crude solid was purified by silica gel column chromatography using ethyl acetate : n-hexane = 1:4 as eluent. Then, the solvent was evaporated using rotary evaporator and dried at room temperature under vacuum for 48 h to give off product. (with powder, yield 69.4%); δ_H(400MHz, $CDCl_3$) 6.23(2H, s, H_a), 3.20(2H, s, H_b), 2.46(2H, s, H_e), 2.38(6H, s, H_f), 2.09(3H, s, H_h), 1.68(6H, q, *J*=16Hz, H_g), 1.43(1H, d, *J*=8Hz, H_c) 1.29(1H, d, *J*=8Hz, H_d); ATR-IR (cm^{-1}) 2912-2852(C-H stretching), 1760(imide C=O asymmetric stretching), 1684(imide C=O symmetric stretching), 1456(CH_2 deformation), 1372(C-H deformation), 1332(=CH deformation), 1200(C-N stretching), 976(C-C skel.), 780(C=C-H deformation) [1].

S-1-4. Synthesis of Poly(NB-Ad)

For the synthesis of Poly(NB-Ad), 175.1 mg of NB-Ad **(5)**(5.89×10^{-1} mmol) was dissolved in 3 mL of degassed dichloromethane. 5 mg of Grubb's 2nd catalyst (5.89×10^{-3} mmol) was dissolved in 1 mL of degassed dichloromethane followed by added to monomer solution with vigorous stirring at room temperature for overnight. After reaction, excess of ethyl vinyl ether was added to solution for quench the reaction. Solution was purified by precipitating from hexane and dried for 48 h under vacuum at room temperature to give off product. (pale gray powder, yield 72.2%); δ_H(400MHz, $CDCl_3$) 5.69-5.41

(2H, br signal, $H_{a(cis,trans)}$), 3.34-2.56 (4H, br signal, $H_{c,d}$), 2.36 (6H, s, H_e), 2.07(3H, s, H_g), 1.67 (6H, br signal, H_f), 1.19-1.53 (2H, br signal, H_b); ATR-IR (cm^{-1}) 2908-2852(C-H stretching), 1768(imide C=O asymmetric stretching), 1696(imide C=O symmetric stretching), 1452(CH_2 deformation), 1332(=CH deformation), 1200(C-N stretching), 972(C-C skel) [1].

S-2. Characterization and measurements

All the synthesized monomer structure was confirmed via ^1H NMR spectra obtained on an Agilent 400-MR (400 MHz) instrument using $CDCl_3$ as a reference or an internal deuterium lock. For checking functional group in monomer and membrane, the attenuated total reflection fourier transform infrared (ATR-FTIR) spectra were obtained using a Perkin-Elmer spectrum two ATR-FTIR spectrometer in the range of 4000-400 cm^{-1}. The X-ray diffraction patterns(XRD) of the membranes were measured using a Rigaku Smartlab diffractometer operated at a scanning rate of 3 ° min^{-1} in a 2θ range from 5 ° to 30 ° with Cu Kα1 X-ray radiation (λ = 0.15406 nm). The *d*-spacings were calculated using Bragg's law ($d = \lambda/2\sin\theta$). To observe the surface property, scanning electron microscope (SEM) images and energy dispersive X-ray spectroscopy (EDX) images were obtained without coating before imaging on a JEOL JSM-7800F instrument with a 15.0 kV accelerating voltage and an upper secondary electron detector.

The thermal stability of the membranes was analyzed by thermogravimetric analysis (TGA) measurements conducted on a SCINCO TGA-1000 instrument in the range of 30-800 ℃ at a heating rate of 10 °C min^{-1} under a nitrogen flow. The glass transition temperature (T_g) of each crosslinked membrane was measured using a Perkin-Elmer Pyris-1 DSC from -80 ℃ to 150 ℃ with a heating rate of 10 ℃ min^{-1} under a nitrogen flow.

The gel fraction of the crosslinked membrane was measured by immersion in chloroform for 24 hours. The membrane was then dried at 40 °C. for 24 hours. Subsequently, the gel fraction was determined by the following formula by weight before and after extraction (1) :

$$\text{Gel fraction (\%)} = \frac{W_1}{W_2} \times 100 \quad (1)$$

Where, W_1 is the membrane weights before gel fraction (g) and W_2 is the membrane weights after gel fraction (g), respectively.

For the measurement of membrane density, samples were weighed in air and in a HPLC grade high-purity heptane at room temperature using the buoyancy method based on Archimedes principle. The corresponding membrane density was calculated below the equation (2) :

$$\rho_{membrane} = \frac{W_{air}}{W_{air}\text{-}W_{hepatne}} \times \rho_{heptane} \quad (2)$$

Where, $\rho_{membrane}$ is density of membrane (g/cm^3), W_{air} is the membrane weights in air (g), $W_{heptane}$ is the membrane weights in heptane (g), is density of $\rho_{heptane}$ (g/cm^3) respectively.

S-3. Gas permeability measurements

Gas permeability measurements of pure gas were performed using a vacuum applied time-lag instrument based on a constant-volume/variable-pressure method. All of experiments were performed at 2 atm of upstream pressure at 30 °C. Before taking these measurements, both upstream and downstream were thoroughly evacuated to below 10-5 Torr until the readout showed zero values for the removal of any residual gases. The downstream volume was calibrated using a Kapton membrane and was found to be 57.55 cm^3. Both side pressures were measured using a Baratron transducer (MKS; Model No. 626B02TBE) with a full scale of 10,000 and 2 Torr, respectively. The permeability coefficient was calculated from the linear slope of the downstream pressure-time plot (dp/dt) according to the following equation (3):

$$P = \frac{273\ K}{76\ cmHg} \times \frac{Vl}{ATp_0} \times \frac{dp}{dt} \tag{3}$$

where, P is the permeability expressed in Barrer (1 Barrer = 10^{-10} $cm^3(STP)\cdot cm\cdot cm^{-2}\cdot s^{-1}\cdot cmHg^{-1}$), A is the effective area of the membrane (cm^2), V is the downstream volume (cm^3), T is the measurement temperature (K), l is the membrane thickness (cm), p_0 is the pressure of the feed gas in the upstream chamber (Torr), and dp/dt is the rate of the pressure change under a steady state. For single gas, the permeability tests were repeated more than three times, and the standard deviation from the mean values of the permeabilities was within ca. ± 3%. Sample-to-sample reproducibility was high and within ± 3%. The effective membrane areas were 15.9 cm^2. The ideal permselectivity, $\alpha_{A/B}$, of the polymeric membrane for a pair of gases (A and B) is calculated as the ratio of the individual gas permeability coefficients.

The diffusivity coefficient was obtained from the time-lag (θ) value according to the equation (4):

$$D = \frac{l^2}{6\theta} \tag{4}$$

where, D is the diffusivity coefficient ($cm^2\cdot s^{-1}$), l is the membrane thickness (cm) and θ is the time lag (s), as obtained from the intercept of the linear steady-state part of the downstream pressure-time plot.

Since the permeability coefficient (P) is product of diffusivity coefficient (D) and solubility coefficient (S), solubility coefficient was calculated from equation (5) with the permeability and diffusivity coefficient obtained from Equations (3) and (4).

$$S = \frac{P}{D} \tag{5}$$

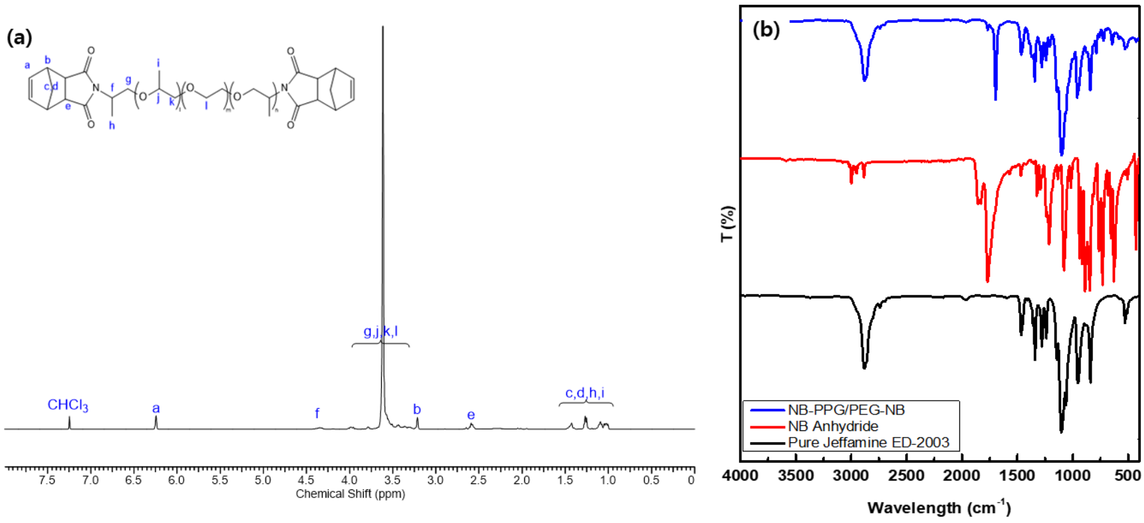

Figure S1. (a) ^{1}H NMR spectrum and (b) ATR FT-IR spectra of the NB-PPG/PEG-NB **3**

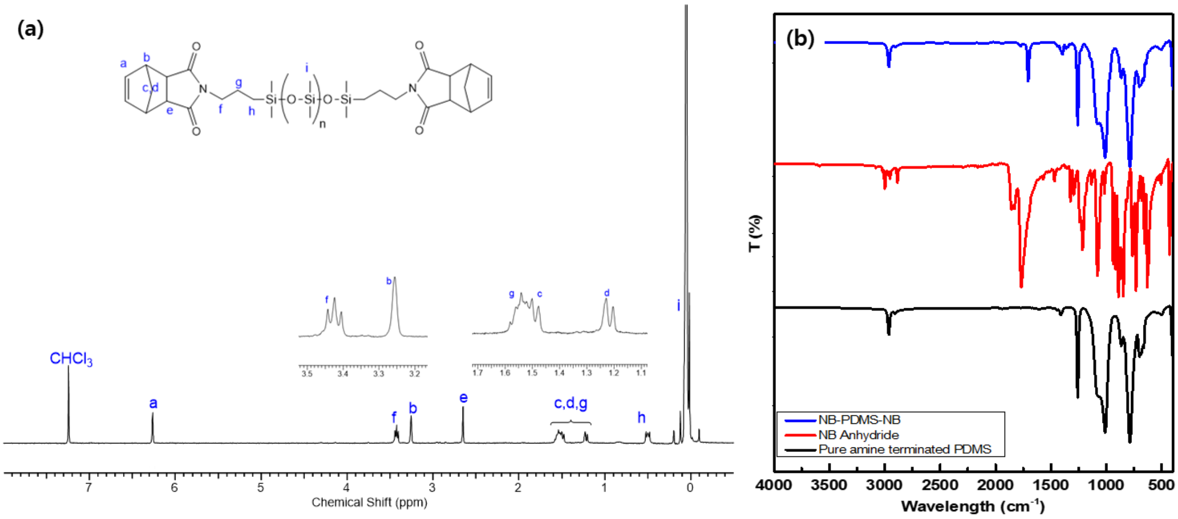

Figure S2. (a) ^{1}H NMR spectrum and (b) ATR FT-IR spectra of NB-PDMS-NB **4**

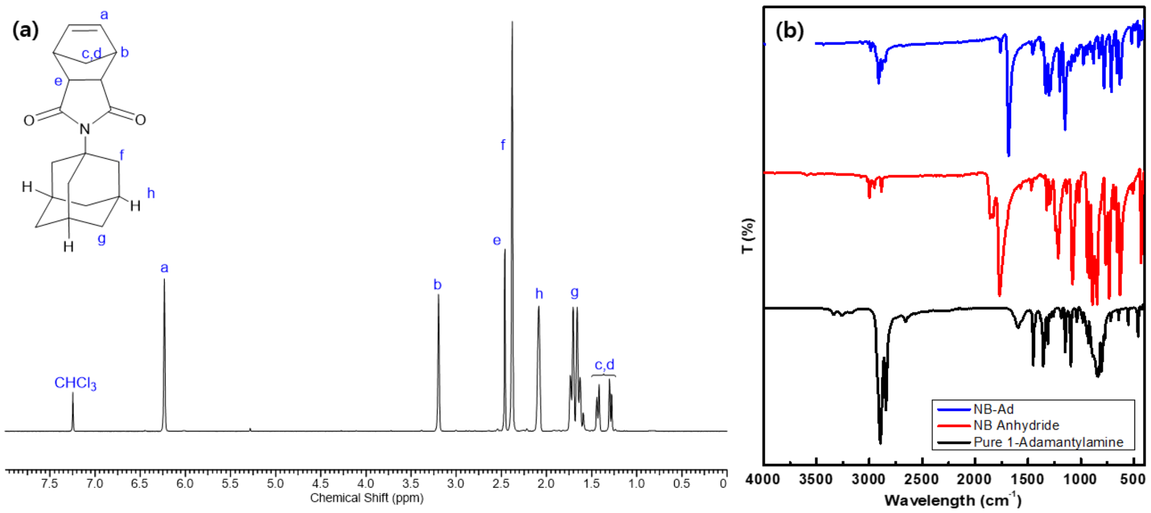

Figure S3. (a) ^{1}H NMR spectrum and (b) ATR FT-IR spectra of NB-Ad **5**

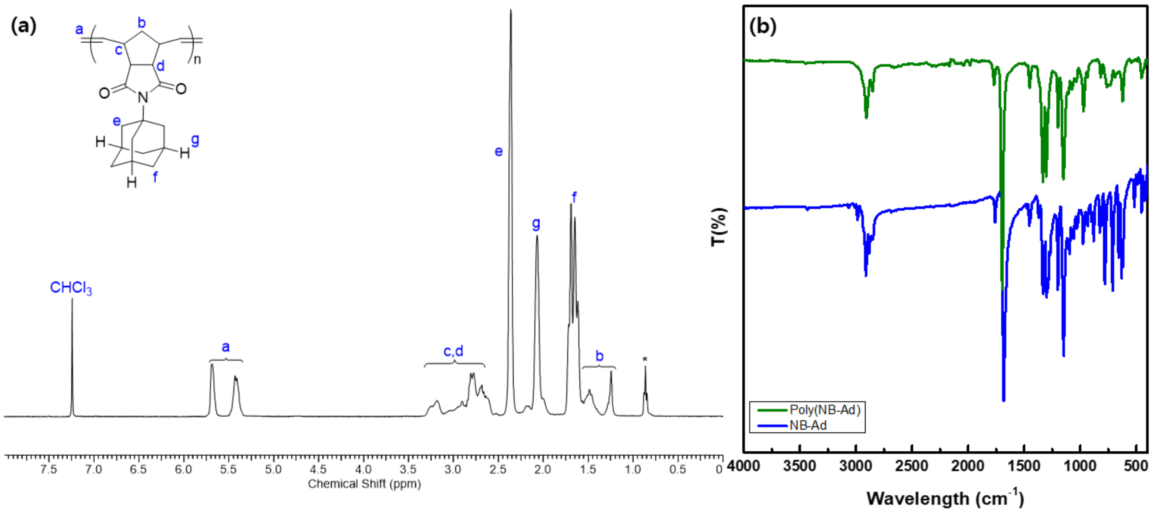

Figure S4. (a) ^{1}H NMR spectrum and (b) ATR FT-IR spectra of Poly(NB-Ad)

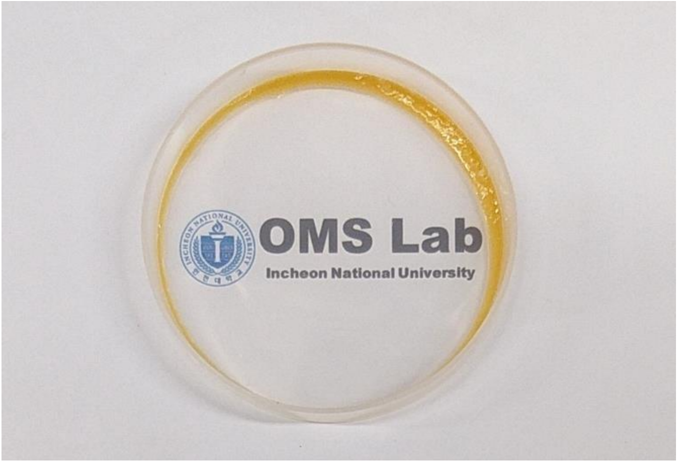

Figure S5. Photograph of the control membrane that was made solely of precursors and dried without the addition of Grubbs 2nd catalyst.

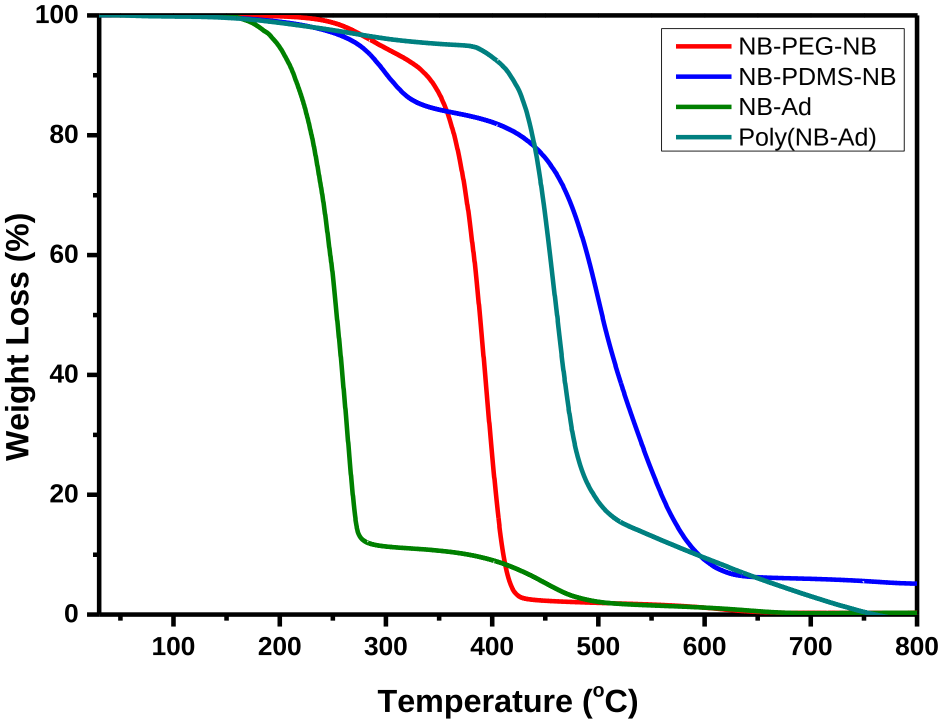

Figure S6. TGA curves of the three precursors used for polymerization and the Poly(NB-Ad) homopolymer at 30-800 ℃

Table S1. Gas transport properties of the crosslinked copolymer membrane previously developed, x(PEG/PPG:PDMS)(1:0.10) membrane [2].

x(PEG/PPG:PDMS)(1:0.10)	Gas molecule			Selectivity (α)	
	CO_2	N_2	CH_4	CO_2/N_2	CO_2/CH_4
Permeability (Barrer)	437.02	7.78	25.16	56.2	17.37
Diffusivity (D)	98.8	20.1	36.4	4.92	2.71
Solubility (S)	4.424	0.387	0.691	11.43	6.40

Reference

1. Pineda Contreras, A.; Tlenkopatchev, M.A.; Del Mar López-González, M.; Riande, E. Synthesis and gas transport properties of new high glass transition temperature ring-opened polynorbornenes. *Macromolecules* **2002**, *35*, 4677–4684.
2. Hossain, I.; Kim, D.; Al Munsur, A.Z.; Roh, J.M.; Park, H.B.; Kim, T.-H. PEG/PPG–PDMS-Based Cross-Linked Copolymer Membranes Prepared by ROMP and In Situ Membrane Casting for CO 2 Separation: An Approach to Endow Rubbery Materials with Properties of Rigid Polymers . *ACS Appl. Mater. Interfaces* **2020**, *12*, 27286-27299.
